# Candida Albicans Lung Abscess in an Illicit Drugs User With Hepatitis C Virus Chronic Infection

**DOI:** 10.7759/cureus.13117

**Published:** 2021-02-04

**Authors:** Maddalena Zippi, Antonella Toma, Francesca Maccioni, Roberta Pica

**Affiliations:** 1 Unit of Gastroenterology and Digestive Endoscopy, Sandro Pertini Hospital, Rome, ITA; 2 Unit of Urgent Digestive Endoscopy, Sandro Pertini Hospital, Rome, ITA; 3 Department of Radiological Sciences, Oncology and Pathology, Sapienza University, Policlinico Umberto I, Rome, ITA

**Keywords:** candida albicans, cocaine, hepatitis c virus, illicit drugs, lung abscess

## Abstract

Illicit substances are widely used all over the world. Among them, crack cocaine results to be the most used drug for the fact that it can be taken in different ways, such as inhaled or intravenous. Pulmonary complications are well known in people snorting it, mostly due to contamination with other substances contained in the objects able to infuse the drug. Herein, we present a case of lung candida abscess related to nasal insufflation of cocaine in an abuser patient suffering from hepatitis C virus (HCV) and not immunocompromised.

## Introduction

Candida albicans lung abscess is a rare occurring medical event [1]. The consumption of illicit drugs has been associated with several pulmonary complications, including pneumonia, pulmonary hemorrhage, cardiogenic edema, and acute lung injury [2]. Generally, these consequences are the result of chronic cocaine use, which can occur through different routes of administration, viz. orally, intranasally, intravenously, or by inhalation [2]. Crack cocaine, a thick smokable solid, appeared on the market during the second half of the 1980s and today it represents one of the most widely used substances for this characteristic [3]. Its new formulation is also injectable [4].

To date, from an examination of the available literature, no case of lung abscess, in specific supported by Candida albicans, has been portrayed in illicit substance abuser patients suffering from hepatitis C virus (HCV) and not immunocompromised.

## Case presentation

A 36-year-old prisoner man was admitted to our hospital due to a two-week history of fever (38-39°C), asthenia, cough, and hemoptysis, not responding to antibiotic therapy. His clinical history exposed his habitual consumption of smoked crack cocaine and injected heroin, as well as untreated HCV-related liver disease. His vital signs included a blood pressure of 130/80 mmHg, heart rate of 90 beats/minutes, respiratory rate of 16 beats/minutes, and oxygen saturation of 95% in room air. The lung auscultation highlighted the presence of crackles at the right base of the organ.

**Figure 1:**
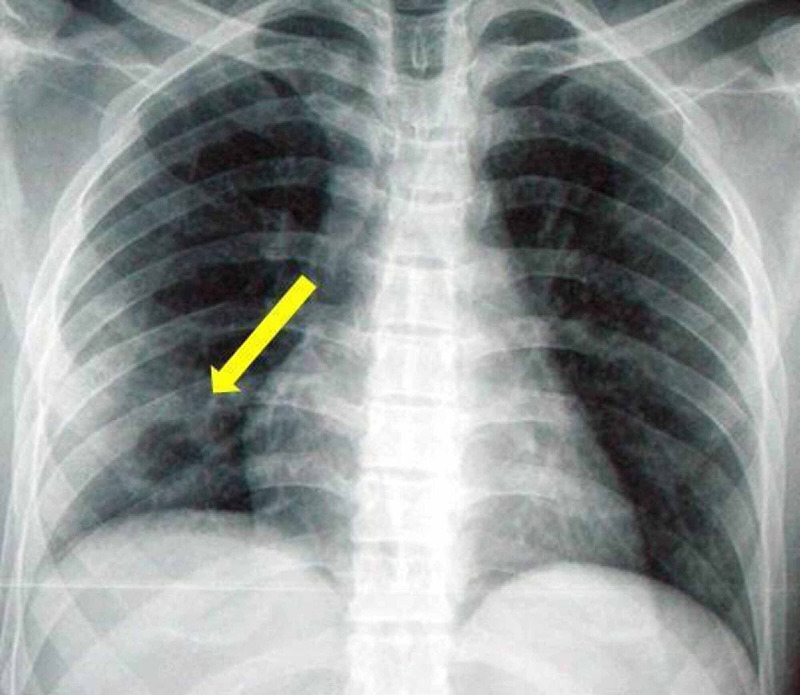
Chest X-ray showed a round and excavated lesion (arrow).

**Figure 2:**
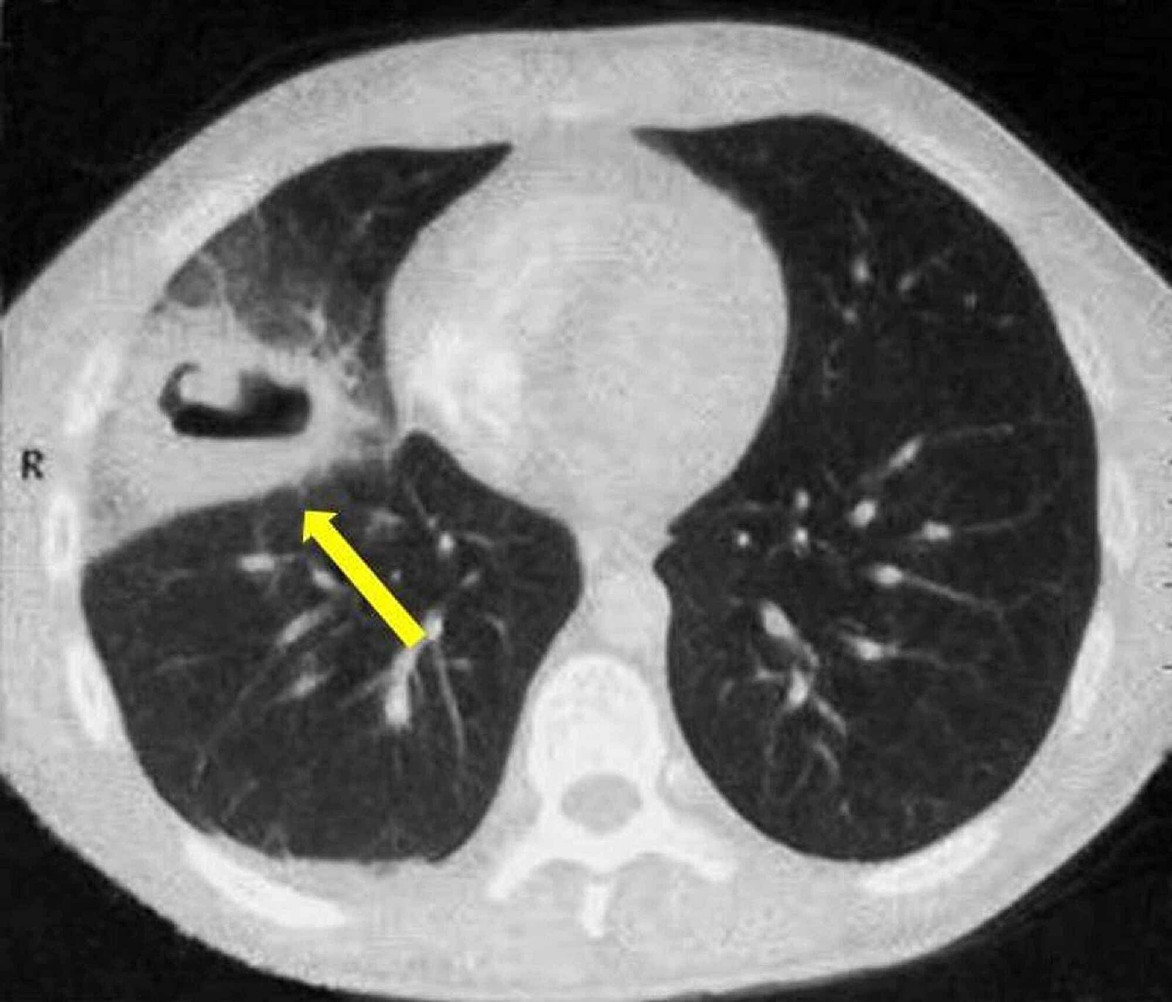
Chest-CT with contrast agent, axial view, showing a rounded lesion with a thick wall (arrow) in the right lower lobe.

Increases in white blood cell count (14,160/mm3; neutrophils, 77%) and index of inflammation (erythrocyte sedimentation rate, three-fold and protein C-reactive, five-fold) were present. The other laboratory findings resulted to be normal and there was no sign of immunodeficiency. The serological tests for HBV and HIV, as well as the CD4+ T cell count, were negative, whereas the HCV antibody test was positive and the quantitative HCV-RNA was 6.0 log10 IU/ml (5.6-7.4 log10 IU/ml). A chest X-ray showed a 7 cm round and excavated lesion in the right lower lobe (Figure 1), confirmed by CT performed with a contrast agent, revealing active mediastinal lymph nodes (Figure 2). Quantiferon-TB Gold (QFT), tuberculin skin testing, and sputum examination for acid-fast bacilli (AFB) were negative. Blood cultures and tumor markers were negative too. Due to persistent respiratory symptoms, bronchoscopy with bronchoalveolar lavage (BAL) was carried out and its cytological sample showed the suspicion of an ongoing Candida albicans infection, without evidence of tumoral cells or mycobacterium tuberculosis. Candida infection was confirmed by three consecutive positive respiratory sputum cultures. During hospitalization, the patient’s liver disease was also studied by a Fibroscan test, obtaining a score for hepatic fibrosis of F2, with a genotyping resulting in type 3. The diagnosis of Candida lung abscess was suggested. Treatment with intravenous fluconazole 400 mg daily was promptly started, later replaced by its oral formulation, and clinical improvement ensued. After three weeks, the antifungal treatment was discontinued and the patient was thus discharged, with a complete recovery. He was given an appointment to be followed at our hepatological unit and to perform the treatment with direct-acting antiviral agents (DAAs) for his chronic hepatitis. After three months of therapy with sofosbuvir 400 mg/velpatasvir 100 mg one time daily, the sustained virological response (SVR) was reached and it’s still active.

## Discussion

Lung abscess due to infection of Candida spp. is an extremely rare event. During the past years, very little has been published on the relationships between Candida albicans and lung abscess [1]. In 60% of cases, primary lung abscesses are identified, ie., when underlying pulmonary diseases are found, frequently due to the aspiration of oral material, poor oral cleanliness, dental infections, alcohol abuse, and chronic illicit drug use [5]. In the remaining 40% of cases, secondary abscesses are registered, also due to vascular emboli [5]. Pre-existing conditions like pulmonary tuberculosis, bronchogenic carcinoma, bronchial asthma, irradiation treatment, malignancy, diabetes mellitus, and malnutrition make the lungs susceptible to be invaded by the candida species [6]. Also, long term antibiotics and steroids therapy was found to be associated with pulmonary candidiasis [6]. Candida spp. are frequently cultured from respiratory secretions owing to the contamination with the oral flora [7]. Generally, the isolation of Candida spp. from BAL is not enough and the diagnosis of its infection requires the confirmation of tissue invasion from biopsy specimen by histopathological studies [7,8]. In our case, we did not carry out a CT guided percutaneous needle aspiration of the lesion, as the patient quickly responded to the antifungal therapy. However, on the basis of CT images, a superinfection supported by Aspergillus spp. was also taken into account. Although the three consecutive respiratory sputum cultures were not tested positive to the presence of this pathogen and it was not found in BAL either, the eventuality of an occurring coinfection was not totally ruled out. In any case, the undertaken therapy would have been able to eradicate also this kind of fungal disease. Recently, as underlined by Randhawa et al., polymerase chain reaction (PCR) for specific nucleotide sequences of this pathogen allows to identify it in cultures of urine, blood, and respiratory tract [9]. Generally, addicts use two-to-three drops of lemon juice to dilute and to increase the solubility of heroin and crack cocaine, previously processed with ammonia or sodium bicarbonate, before injecting them intravenously [10,11]. It has been shown how lemon juice, used for this purpose, can promote the appearance of abscesses, permanent damage to the veins, and infections [12]. It has also been speculated that the lemon may have been contaminated by heroin abusers themselves, as disseminated candidiasis, supported by Candida albicans, occurred in three cases in which this fruit was left at room temperature for a week and then reused [13]. It has been reported how the assumption of cocaine through nasal insufflation can determine the appearance of pulmonary complications, among which also abscesses [14]. Two mechanisms have been hypothesized to play a role in their onset: the first one is the direct inhalation of pathogens contaminating the substance, the second one is the direct action of cocaine on alveolar macrophages, resulting in an inhibited response by leukocytes [14]. In this regard, a study conducted on animal models from Jayaraja et al. is interesting [15]. The researchers, after having intratracheally instilled Candida albicans in mice, performed an analysis on the bronchoalveolar lavage, which contained macrophages alveolar, and neutrophils [15]. Afterward, they noted how cytosolic phospholipase A2 (cPLA2α), which is activated in response to this specific pathogen, with consequent resulting in the release of arachidonic acid essential for the production of eicosanoids, plays a key role in the immune host system against the commensal organism Candida albicans [15]. Finally, several extrahepatic manifestations (EMs) were described in patients with HCV, but the only one recognized involving the respiratory system is idiopathic pulmonary fibrosis (IPF) [16]. Our patient suffered from an untreated active chronic hepatitis C and, for this reason, he was not immunosuppressed by any drug.

## Conclusions

The diagnosis of lung abscess has to be affirmed starting from an initial differential diagnosis of other pathologies, including infectious diseases. Candida albicans can determine the onset of this pulmonary complication, especially in subjects habitually users of inhaled cocaine contaminated with this pathogen. This case underlines the importance of alerting physicians to this complication, which can occur in drug users, even in those not immunosuppressed.
